# The effects of *Bacillus subtilis *on* Caenorhabditis elegans *fitness after heat stress

**DOI:** 10.1002/ece3.4983

**Published:** 2019-02-18

**Authors:** Kim L. Hoang, Nicole M. Gerardo, Levi T. Morran

**Affiliations:** ^1^ Department of Biology Emory University Atlanta Georgia

**Keywords:** beneficial microbes, context‐dependency, environmental stress, host–microbe association

## Abstract

Microbes can provide their hosts with protection from biotic and abiotic factors. While many studies have examined how certain bacteria can increase host lifespan, fewer studies have examined how host reproduction can be altered. The nematode *Caenorhabditis elegans *has been a particularly useful model system to examine how bacteria affect the fitness of their hosts under different contexts. Here, we examine how the bacterium *Bacillus subtilis*, compared to the standard *C. elegans *lab diet, *Escherichia coli*, affects *C. elegans *survival and reproduction after experiencing a period of intense heat stress. We find that under standard conditions, nematodes reared on *B*. *subtilis* produce fewer offspring than when reared on *E*. *coli*.However, despite greater mortality rates on *B*. *subtilis* after heat shock, young adult nematodes produced more offspring after heat shock when fed *B*. *subtilis* compared to *E. coli*. Because offspring production is necessary for host population growth and evolution, the reproductive advantage conferred by *B*. *subtilis* supersedes the survival advantage of *E. coli*. Furthermore, we found that nematodes must be reared on *B*. *subtilis* (particularly at the early stages of development) and not merely be exposed to the bacterium during heat shock, to obtain the reproductive benefits provided by *B*. *subtilis*. Taken together, our findings lend insight into the importance of environmental context and interaction timing in shaping the protective benefits conferred by a microbe toward its host.

## 1 INTRODUCTION

Eukaryotic hosts generally obtain fitness benefits through association with specific microbes. Harboring certain microbes can increase host protection from biotic and abiotic stresses, such as enemies or environmental changes, and can provide hosts with nutrients that they cannot obtain from their diet alone (Douglas, 2009; Feldhaar, 2011; Oliver, Smith, & Russell, 2013). Associating with such beneficial microbes can shape host evolution, altering host maintenance of redundant traits (Martinez et al., 2016), and can lead to niche expansion, allowing hosts to occupy environments they normally would not be able to inhabit (Douglas, 2014; McFall‐Ngai et al., 2013). Host–microbe associations are often context‐dependent such that benefits are associated with harboring microbes only under certain conditions, and costs are revealed under others (Heath & Tiffin, 2007; Russell & Moran, 2006; Weldon, Strand, & Oliver, 2013). Additionally, different microbial species, or even strains of the same species, can confer different levels of benefits to hosts of the same genotype, and hosts of different genotypes may also differ in the level of benefit that they receive from microbial association (Murfin et al., 2015; Parker, Hrček, McLean, & Godfray, 2017). Taken together, the hosts, the microbes, and the environment all shape the nature of these interactions over ecological time, which in turn may shape the evolutionary trajectories of the host and microbial populations. Here, we utilize *Caenorhabditis elegans*, a well‐characterized invertebrate model amenable to a range of experimental manipulations, to test the effects of environmentally obtained bacteria on host fitness under stress.

**Figure 1:**
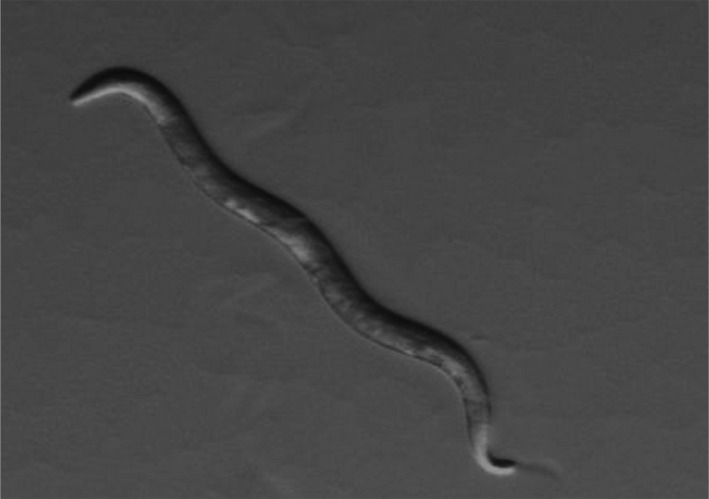
An adult *Caenorhabditis elegans*. Photo credit: McKenna Penley

The nematode *C. elegans *has been extensively used as a model system to study host–microbe associations (Clark & Hodgkin, 2014; Kurz & Ewbank, 2000; Zhang et al., 2017). *Caenorhabditis elegans* (Figure 1) has a natural interaction with microbes in that it feeds on bacteria and fungi in decomposing plant matter (Frézal & Félix, 2015). The nematode has a grinder in the pharynx region that crushes microbes that it consumes; however, some microbes survive the grinder and colonize the gut of the nematode (Gibson et al., 2015; Portal‐Celhay & Blaser, 2012). Some of these persistent microbes are pathogenic to the host (Couillault & Ewbank, 2002), some are commensal (Clark & Hodgkin, 2014), and some are beneficial (Zhang et al., 2017). Specifically, some microbes have been shown to increase nematode lifespan under environmental stresses (Donato et al., 2017; Grompone et al., 2012; Gusarov et al., 2013; Leroy et al., 2012; Nakagawa et al., 2016), an important finding given that *C. elegans* is a model system to study longevity and aging (Cabreiro & Gems, 2013; Garigan et al., 2002; Johnson, 2008). Two studies found that the soil bacterium, *Bacillus subtilis*, was able to increase *C. elegans* survivorship after heat shock relative to exposure to the standard lab diet,* Escherichia coli *(Donato et al., 2017; Gusarov et al., 2013). These studies found that *B*. *subtilis* nitric oxide (NO) production and biofilm formation in the host's gut resulted in elevated host lifespan postheat stress. For this interaction to impact host–microbe evolution, the bacteria would not only need to increase survival but would also need to increase host reproduction after heat shock. *Caenorhabditis elegans* generally exhibit little to no fecundity after exposure to intense heat stress (Aprison & Ruvinsky, 2014), and it is unclear if interactions with *B*. *subtilis* could mitigate this substantial fitness loss. In this study, we measure the effects of *C. elegans* interactions with *B*. *subtilis* on nematode fitness (encompassing both survival and fecundity) after a stressful heat event. Additionally, we determine how exposure to *B*. *subtilis* at different time points during development affects *C. elegans* fitness.

## 2 MATERIAL AND METHODS

### 2.1 Nematode and bacterial strains

All nematodes in this study were the *C. elegans *N2 Bristol strain, which were maintained on *E. coli *OP50 prior to experiments. We used *B*. *subtilis* 168 and *E. coli *OP50 in all experiments. In the first two experiments, we also used *B*. *subtilis* Δ*nos*, which lacks the ability to produce NO. We obtained both *B*. *subtilis* strains from the study examining the role of NO in *C. elegans *longevity and survivorship postheat stress (Gusarov et al., 2013) . We grew both *B*. *subtilis* strains and *E. coli *on nematode growth medium (NGM) plus glucose (2%) and arginine (0.5 mM) for all experiments (Gusarov et al., 2013). For experiments involving fecundity, we transferred nematodes to GFP‐labeled OP50 (OP50‐GFP, grown on NGM) to allow them to produce offspring. We used OP50‐GFP to control for the bacterium that nematodes were exposed to during heat shock recovery. GFP‐labeled *E. coli *is different from *E. coli *OP50 but is still relatively neutral with respect to *C. elegans *fitness.

### 2.2 Survival of 6‐day‐old nematodes on *B*. *subtilis* and *E. coli*

We first compared the short‐term survivorship over the 6 hr postheat stress of nematodes across *B*. *subtilis* 168, *B*. *subtilis* Δ*nos*, and *E. coli *by performing the heat shock experiment done in the previous studies examining the role of *B*. *subtilis* on host lifespan after heat stress (Donato et al., 2017; Gusarov et al., 2013) . We surface sterilized *C. elegans *N2 eggs using an established alkaline hypochlorite protocol (Stiernagle, 2006) and reared L1 larvae on *E. coli *until they reached L4/young adult (on day 3). We then transferred nematodes to either *B*. *subtilis*, *B*. *subtilis* Δ*nos*, or *E*. *coli*.When nematodes were 5 days old, we transferred them to new plates of the appropriate bacteria to prevent mixing of generations. Prior to heat shock, all nematodes were kept at 20°C. On the next day, when they were 6‐day‐old adults, we heat shocked the nematodes in an incubator set at 34°C. After 3 hr, we removed a set of replicate plates for each bacterial treatment from the incubator and scored survival by prodding with a platinum pick to determine signs of movement (Donato et al., 2017; Gusarov et al., 2013; King et al., 2016) . After 6 hr, we removed another set of plates and scored survival. There were three replicate populations per bacterium per time point, each population containing about 20 nematodes.

### 2.3 Survival and fecundity of 3‐day‐old nematodes on *B*. *subtilis* and *E.coli*

Because nematodes cease egg production after about 6 days (Altun & Hall, 2009), to assess fitness effects of *B*. *subtilis* association, here, we heat shocked nematodes when they were young adults and still capable of producing offspring. Specifically, we investigated 3‐day‐old nematode survival and fecundity under standard and heat shock conditions on *B*. *subtilis*, *B*. *subtilis* Δ*nos, *and *E. coli*. We surface sterilized *C. elegans *N2 eggs and reared L1 larvae on each bacterium for 3 days at 20°C until they reached young adulthood. We then placed nematodes in an incubator set at either 20°C (standard temperature) or set at 34°C for 6 hr. We used three replicate populations per bacterium per temperature setting, for a total of 18 populations with approximately 200 nematodes per population. To measure survival, here, and in all subsequent experiments, we determined the proportion of nematodes that were alive 6 hr after the heat shock period (based on prodding, as above), not how long they lived after heat shock (i.e., survivorship or lifespan). Afterward, we washed nematodes from each replicate population with M9 and transferred all adults from each population to another plate seeded with *E. coli *OP50‐GFP to produce offspring, where they were maintained at 20°C. Two days postheat shock, we counted the larvae on each plate. The total number of adults was counted prior to heat shock. Since all plates were transferred using the same protocol, we assumed a similar number of nematodes were transferred to OP50‐GFP to produce offspring. We did not count the number of live adults 2 days postheat shock because nematodes could have produced offspring and subsequently died before being counted. We calculated the average number of offspring per heat shocked adult to determine relative differences in fecundity between treatments. This measure accounts for both difference in survival and difference in fecundity.

### 2.4 Assaying importance of exposure window, experiment 1

Similar to the survival and fecundity assay described above, we surface sterilized N2 eggs and reared L1 larvae on either *B*. *subtilis* or *E. coli *for 3 days, with six replicates per bacterium. We then transferred three *B*. *subtilis* replicates to *E. coli *and the other three replicates to *B*. *subtilis*, and similarly transferred the *E. coli *replicates to either *E. coli *or *B*. *subtilis*, for a total of 12 populations with approximately 100 nematodes per population. We heat shocked nematodes at 34°C for 6 hr, scored survival after 6 hr as above, then transferred them to plates seeded with *E. coli *OP50‐GFP to lay eggs, where they were maintained at 20°C. We quantified larval offspring 2 days later.

### 2.5 Assaying importance of exposure window, experiment 2

**Figure 2:**
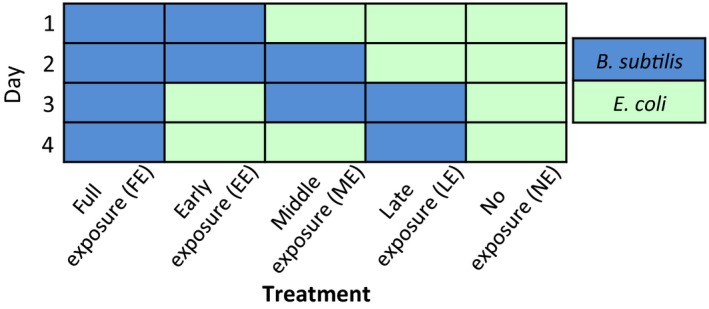
Setup of exposure experiment 2. Nematodes were transferred to the indicated bacterium on each day and heat shocked on day 4

Similar to the first exposure assay, we surface sterilized N2 eggs and transferred about 100–200 larvae to a new lawn of bacteria each day as indicated in Figure 2 (with four replicates per treatment, for a total of 20 populations). After reaching adulthood, we heat shocked nematodes for 6 hr at 34°C, scored survival after 6 hr as above, then transferred nematodes to plates seeded with *E. coli *OP50‐GFP to lay eggs, where they were maintained at 20°C. We quantified larval offspring 2 days after.

### 2.6 Colonization of day 2 larvae and adult nematodes

Following a modified protocol from Vega & Gore (2017), we determined whether day 2 larvae reared on *B*. *subtilis* harbored live bacterial cells. Briefly, after surface sterilizing N2 eggs, we transferred roughly 100 larvae to a lawn of *B*. *subtilis*. The following day (day 2 of Figure 2), we washed larvae three times with cold 0.01% Triton X‐100 in M9 and incubated them in bleach (1:1,000 diluted) for 15 min at 4°C to remove surface bacteria. We then treated larvae with a solution of 0.25% sodium dodecyl sulfate (SDS) + 3% dithiothreitol (DTT) for 20 min. After washing with 0.01% Triton X‐100 in M9, we transferred about 10–20 larvae to a well of a 96‐well plate (five wells total), each well containing a small amount of sterile silicon carbide grit and 0.01% Triton X‐100 in M9. We briefly disrupted the samples using a Qiagen TissueLyser II homogenizer. After plating the content onto LB plates, we grew the bacteria for 2 days before quantifying colony‐forming units. For colonization of adults, we reared surfaced‐sterilized N2 eggs on *B*. *subtilis* until adulthood, then heat shocked nematodes for 6 hr at 34°C. We subsequently washed and homogenized the nematodes using the same protocol as the day 2 larvae, crushing five adults in each of five wells of the 96‐well plate.

### 2.7 Statistical analysis

To analyze short‐term survivorship of 6‐day‐old hosts under different bacterial treatments, we used a Cox proportional hazards model with the Coxph function of the Survival package in R (Therneau & Grambsch, 2000). For subsequent experiments, we analyzed survival using a generalized linear model (GLM) with a binomial distribution and logit link function. For fecundity, we used a GLM with a normal distribution and identity link function. We then performed contrast tests to compare bacterial treatments. We used JMP Pro (v.13) for the GLM analyses.

## 3 RESULTS

### 3.1 *Bacillus subtilis* differentially affects survival of old and young adult hosts and provides a reproductive benefit in young adult *C. elegans*

**Figure 3:**
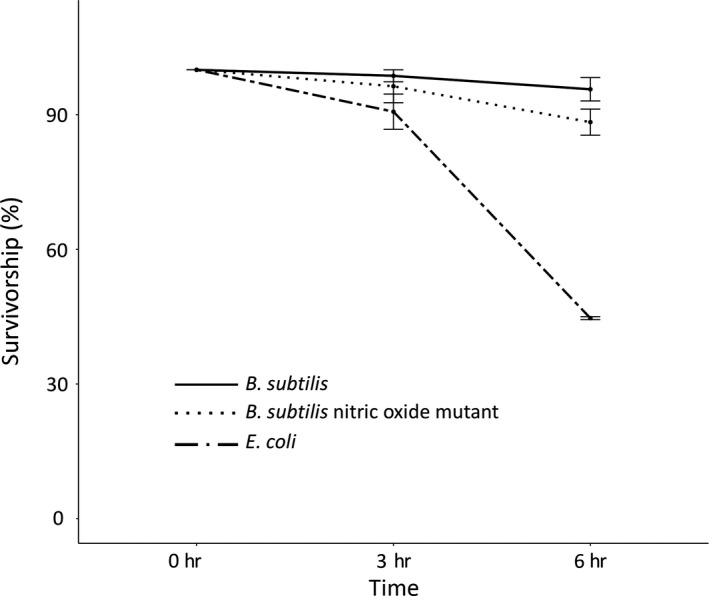
Survivorship of older adult (6‐day‐old) nematodes under heat shock. Nematodes were reared on *Escherichia coli* OP50 until L4/young adult stage (at about 3 days old), then subsequently transferred to the indicated bacterium. They were heat shocked at 34°C 3 days later. After 3 and 6 hr, replicate plates were removed from the heat and scored for survival. Error bars indicate standard errors. There were three replicate populations per time point per bacterium, each population containing ~20 nematodes

We performed a heat shock experiment, similar to previous studies (Gusarov et al., 2013), that allowed us to directly compare wild‐type *B*. *subtilis* strain 168, a *B*. *subtilis* mutant lacking the ability to produce NO (*B*. *subtilis* Δ*nos*), and *E. coli *strain OP50. Briefly, we reared nematodes on *E. coli *at 20°C (standard temperature) for 3 days, then transferred them to one of the three bacterial strains, where they remained for another 3 days before shifting to 34°C (heat shock temperature) for 6 hr. We found that there was a small but nonsignificant decrease in host survival when nematodes were exposed to *B*. *subtilis* Δ*nos* compared to hosts exposed to wild‐type *B*. *subtilis* (Figure 3; $\chi_{1}^{2}$ = 3.36, *p = *0.07). However, we found a significantly large difference in host survival between wild‐type *B*. *subtilis* and *E. coli *immediately after 6 hr of heat shock ($\chi_{1}^{2}$ = 28.05, *p < *0.001). Therefore, *B*. *subtilis* conferred greater host survival following heat shock, but the protective effects of NO can, at most, only account for a portion of the benefits conferred by *B*. *subtilis*.

**Figure 4:**
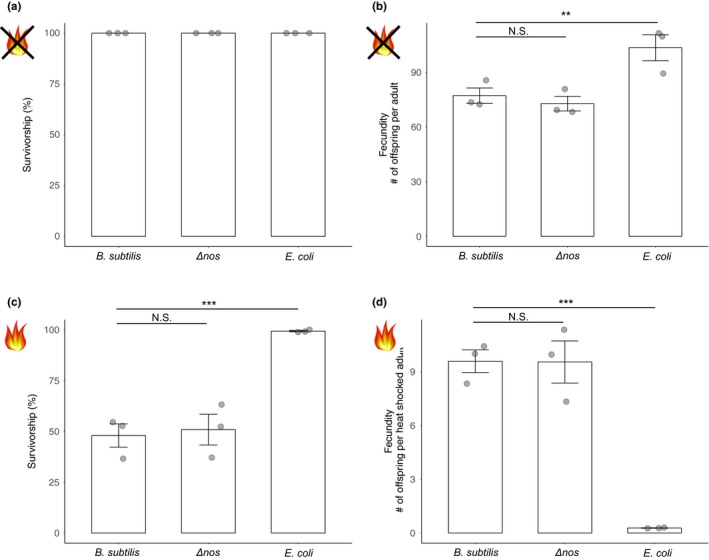
Survival and fecundity of young adult (3‐day‐old) nematodes under no heat shock and heat shock conditions. Nematodes were reared on the indicated bacterium until 3 days old, then were either left at standard conditions or heat shocked. (a) Survival after 6 hr and (b) fecundity of nematodes under standard conditions. (c) Survival after 6 hr and (d) fecundity of nematodes under heat shock conditions. Error bars indicate standard errors. Each data point represents a replicate population, with each population consisting of ~200 nematodes. **p < 0.01, ***p < 0.001, N.S. denotes no significance

Since the nematodes above, as in previous studies, were heat shocked at postreproductive age (egg laying ceases after about 6 days from the time nematodes hatch (Altun & Hall, 2009)), we could not determine whether these bacteria affected host fecundity after heat shock. To this end, we examined the survival and fecundity of young adult *C. elegans *when reared on *B*. *subtilis* 168, *B*. *subtilis* Δ*nos*, and *E. coli* OP50 under standard and heat shock conditions. Three‐day‐old nematodes reared on their respective bacterium at 20°C were either left at the standard temperature or heat shocked. We found no difference in host survival under standard conditions (measured at the same time we scored survival of heat shocked nematodes) regardless of the hosts’ bacterial association (Figure 4a). However, nematodes reared on *E. coli *produced more offspring than on either *B*. *subtilis* strain under standard lab conditions (Figure 4b; $\chi_{2}^{2}$ = 13.04, *p = *0.0015). Under heat shock conditions, more nematodes survived on *E. coli *compared to both *B*. *subtilis* strains (Figure 4c; $\chi_{1}^{2}$ = 611.03, *p < *0.001). By contrast, more offspring were produced on both *B*. *subtilis* strains compared to *E. coli *(Figure 4d; $\chi_{1}^{2}$ = 25.53, *p < *0.001). Therefore, *B*. *subtilis* exposure conferred increased fecundity per adult going into heat shock, but not survival, in young adult nematodes. Further, *B*. *subtilis* NO production did not increase the survival of heat shocked, young adult nematodes compared to *B*. *subtilis* Δ*nos* (Figure 4c; $\chi_{1}^{2}$ = 0.89, *p = *0.35) and was not necessary for the increased reproduction conferred by *B. subtilis*. Because we saw no significant differences between the two *B*. *subtilis* strain treatments, all subsequent experiments used only the wild‐type *B*. *subtilis* 168 strain.

### 3.2 Development on *B*. *subtilis* is necessary for reproductive benefit

**Figure 5:**
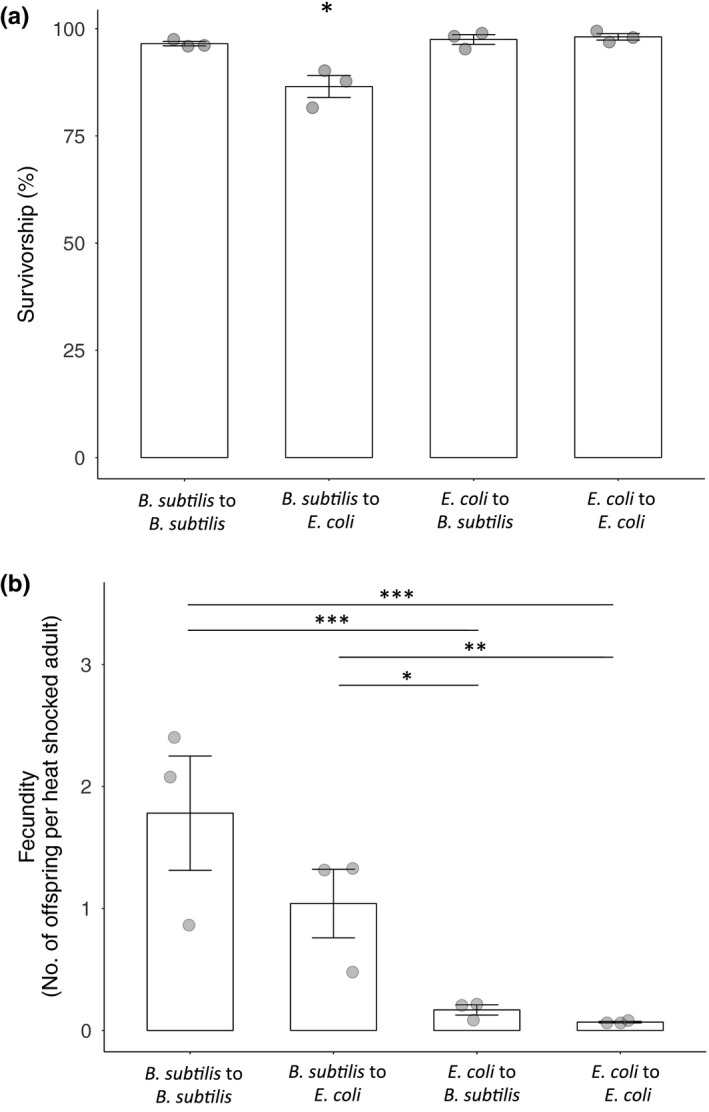
Survival and fecundity of young adult nematodes heat shocked on either *Bacillus subtilis* or *Escherichia coli*. Nematodes were reared on the first bacterium indicated before being transferred to the bacterium they were heat shocked on when they reached adulthood. (a) Survival after 6 hr and (b) fecundity of nematodes under heat shock conditions. Error bars indicate standard errors. Each data point represents a replicate population, with each population consisting of ~100 nematodes. *p < 0.05, **p < 0.01, ***p < 0.001

To determine whether the decrease in survival and increase in offspring production on *B*. *subtilis* was due to host larval development on *B*. *subtilis* or simply due to exposure to the bacterium during heat shock, we compared nematodes reared on *B*. *subtilis* that were then heat shocked on *E. coli*, and vice versa. Nematodes that developed on *B*. *subtilis* and were heat shocked on *E. coli* exhibited the lowest survival (Figure 5a; $\chi_{1}^{2}$ = 97.10, *p < *0.001). However, development on *B*. *subtilis* resulted in higher fecundity after heat shock regardless of which bacterium nematodes were exposed to during heat stress (Figure 5b; $\chi_{1}^{2}$ = 15.96, *p < *0.001). The reproductive benefit conferred by *B*. *subtilis* was therefore predominantly dependent upon the development of hosts on *B*. *subtilis*.

### 3.3 Early exposure to *B*. *subtilis* is more beneficial for hosts than later exposure

**Figure 6:**
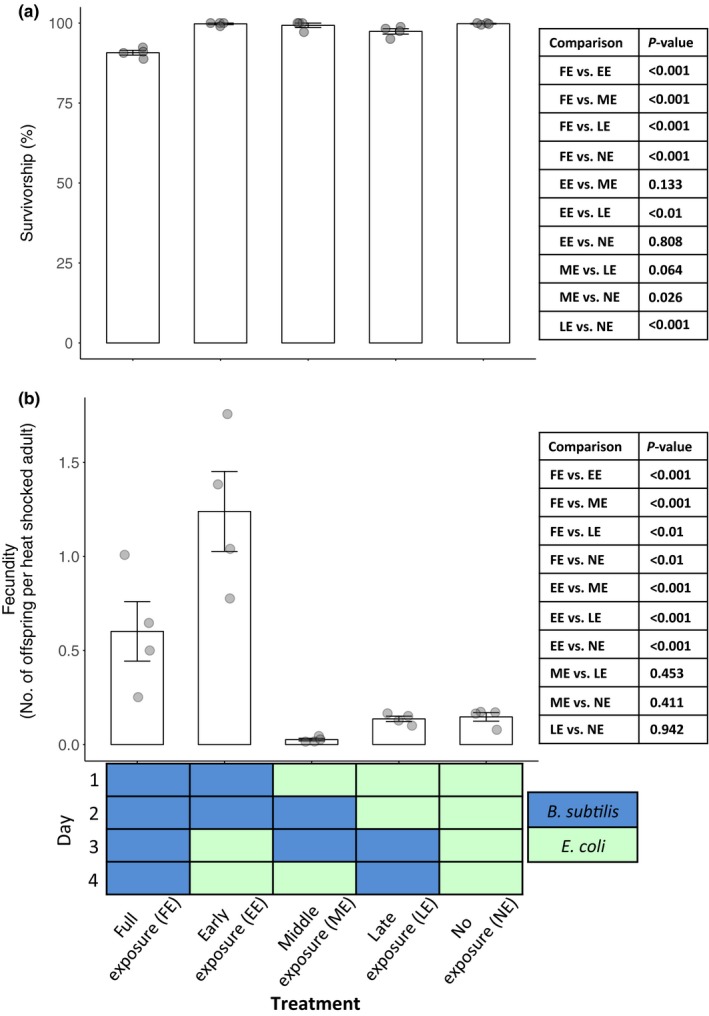
Survival and fecundity of young adult nematodes exposed to *Bacillus subtilis* at different points throughout larval development. Nematodes were transferred to each bacterium as indicated each day before being heat shocked on day 4. (a) Survival after 6 hr and (b) fecundity of nematodes under heat shock conditions. Error bars indicate standard errors. Each data point represents a replicate population, with each population consisting of ~100 to 200 nematodes

We then asked whether the age at which nematodes are exposed to *B*. *subtilis* has an effect on the hosts' survival and fecundity. We varied exposure time to *B*. *subtilis* by transferring nematodes to the indicated bacterium each day (Figure 2). We found that the time at which the host is exposed to *B*. *subtilis* affects both survival and fecundity upon heat stress (Figure 6a; $\chi_{4}^{2}$ = 114.61, *p < *0.001; Figure 6b; $\chi_{4}^{2}$ = 35.02, *p < *0.001). Specifically, exposure to *B*. *subtilis* during the first 2 days of host development is critical for nematodes to obtain the reproductive benefit conferred by *B*. *subtilis* upon heat stress (Figure 6b, early exposure treatment vs. all other treatments). Furthermore, compared to when nematodes were exposed to *B*. *subtilis* throughout development and during heat shock, early exposure to *B*. *subtilis* was more beneficial in terms of fecundity and survival (Figure 6, full exposure vs. early exposure). Overall, these results demonstrate that nematodes gained the most benefits when exposed to *B*. *subtilis* early, whereas later exposure to *B*. *subtilis* conferred no greater benefit than exposure to *E. coli *alone (Figure 6b).

### 3.4 Bacterial colonization of host

Because nematodes benefit the most when either exposed to *B*. *subtilis* completely or early on in development, we asked whether these benefits were associated with live *B*. *subtilis* in the nematode gut. We first extracted and grew *B*. *subtilis* colonies from replicate groups of N2 nematodes reared on *B*. *subtilis* until day 2 of Figure 2, observing means ranging from 0.42–0.84 colony‐forming units, or CFU, per larva. The live colonies we found indicate that *B*. *subtilis* cells are able to enter young host larvae, pass through the grinder intact, and survive in the host gut. In addition, we recovered live *B*. *subtilis* in heat shocked adults (mean of 2 CFU per nematode), showing that live *B*. *subtilis* was present in the host after heat stress.

## 4 DISCUSSION

Here, we evaluated the effects of specific host–microbe interactions on host survival and fecundity after environmental change, via heat shock. Overall, we found that while *C. elegans* interactions with *E. coli* resulted in greater host fitness compared to *B*. *subtilis* under standard conditions (Figure 4b), interactions with *B*. *subtilis* conferred significantly greater host fitness, via increased fecundity, after heat shock (Figure 4d). Reproduction is vital for population growth and evolution in the long term—if an individual does not reproduce, it will have no fitness regardless of whether it survives after heat stress. Here, we demonstrated that, under a scenario of heat shock, survival did not necessarily correlate with fecundity. Rather, hosts of reproductive age had lower survival overall on *B*. *subtilis* compared to *E. coli*, but had greater fecundity on *B*. *subtilis* upon heat shock. Further, in corroboration with previous research, we showed that *C. elegans* can reproduce after several hours of high heat stress (Aprison & Ruvinsky, 2014, 2015). Even though hosts undergo some sperm damage, they can produce viable offspring if allowed sufficient time to recover and reproduce (Aprison & Ruvinsky, 2014). Thus, given this reproduction after heat shock, there is potential for *B*. *subtilis* protection to shape host evolution.

Our survival results for heat shocked older adult nematodes support those of previous studies, in that *B*. *subtilis* led to increased host survivorship compared to *E. coli *(Gusarov et al., 2013; Donato et al., 2017). While we did not find a significant difference between association with the two *B*. *subtilis* strains, we did not extend the heat shock period past 6 hr, where a larger difference in survival may be observed. By contrast, when we heat shocked younger adult nematodes, we found lower survival of hosts reared on *B*. *subtilis* (both wild‐type and NO mutant) compared to *E. coli*. We hypothesize that the survival difference is due to the different age of hosts that were heat shocked: young adults (3‐day‐old nematodes) in our study instead of old adults (6 to 8‐day old nematodes) in the prior studies. Interestingly, the increased survival gained by nematodes under the circumstances of the prior experiments would have had little to no effect on host fitness, which was not measured, as these nematodes were past reproductive age. While we have not identified the mechanisms by which *B*. *subtilis* increases fecundity in young adult nematodes upon heat shock, bacterial NO does not appear to be a critical driver in the reproductive output of these nematodes. Taking the survival and reproduction data together, we see that *B*. *subtilis* can confer a reproductive advantage to *C. elegans *hosts while reducing their survival under heat stress.

Investment in longevity is hypothesized to trade‐off with reproduction (Mukhopadhyay & Tissenbaum, 2007). While we did not measure total host lifespan, our heat shock data suggests that increased survival of *C. elegans* on *E. coli *led to a reproductive cost, the converse of which is true for hosts on *B*. *subtilis*. Furthermore, because nematodes have more offspring on *E. coli *than *B*. *subtilis* in the absence of heat shock (Figure 4b), the interaction between *B*. *subtilis* and *C. elegans *is context‐dependent—hosts incur a cost to harboring *B*. *subtilis* in the absence of heat stress, but benefit reproductively under heightened temperatures. Furthermore, while our heat shock temperature (34°C) is much higher than the range at which *C. elegans *is generally reared (15–25°C), hosts were able to reproduce at a rate at which the population could at least replace itself on *B*. *subtilis*, compensating for the reduced number of surviving adults compared to *E*. *coli*.

Exposure to beneficial microbes during the early stages of host development could be important for host resistance to biotic and environmental stresses during adulthood. For example, a study found that prior diet can affect *C. elegans *preference for harmful *Burkholderia *bacteria (Cooper, Carlson, & LiPuma, 2009). Another study examining the consequences of early exposure to pathogens in *C. elegans *found increased resistance to pathogens and heat stress during adulthood (Leroy et al., 2012). Host fecundity may also differ depending on the bacteria the host is exposed to during development and adulthood. Our study provides support for this phenomenon: exposure to *B*. *subtilis* during early stages of development was enough for *C. elegans *to remain reproductively viable after a period of heat shock (Figure 6b). By contrast, exposure to *B*. *subtilis* as an older larva or adult did not benefit hosts greatly when they underwent heat stress (Figure 5b/6b). This suggests that exposure to the bacterium at an early point during nematode development may be critical in priming the host to respond to heat shock as an adult. The bacterium may have entered nematodes as spores or formed spores upon entrance, and so early exposure to the *B*. *subtilis* may have allowed more time for spores to become vegetative and thus benefit nematodes when they were heat shocked. Furthermore, heat shock on *E. coli *after exposure to *B*. *subtilis* for the first 2 days of development offset the cost of reduced survival when heat shocked on *B*. *subtilis* (Figure 6, early exposure vs. full exposure). The nature of this interaction is unclear, particularly given that the impact of *B*. *subtilis* on *C. elegans* fecundity does not appear to be mediated by NO production.

Given that *B*. *subtilis* can be both a gut colonizer and a food source, the results observed may be due to the effects of nutrition obtained via digestion of *B*. *subtilis*. However, several lines of evidence indicate that the increased fecundity of hosts on *B*. *subtilis* is not likely to be solely from diet alone. First, our colonization result suggests that a small number of *B*. *subtilis* cells can colonize young larvae, and that adults harbor a greater abundance of cells after heat shock. Therefore, *B*. *subtilis* can survive the larval grinder and take up residence in the nematode, as well as persist in adults after heat shock. Because early exposure to *B*. *subtilis* resulted in the greatest number of offspring, we hypothesize that early exposure allowed more *B*. *subtilis* to accumulate inside nematodes, thus leading to greater host fecundity after heat shock. Second, if the results were due to the effects of diet, we would expect that exposure to *B*. *subtilis* for an equal amount of time (Figure 6b, all treatments except for full exposure) should result in similar offspring output, whereas feeding solely on *B*. *subtilis* would lead to the highest benefit obtained. Further, since all host individuals were of the same genotype and were exposed to a homogenous lawn of *B*. *subtilis*, we would expect similar levels of nutrient acquisition among individuals both within and between treatment groups, thus resulting in approximately equivalent levels of fecundity between treatments and replicates. However, we observed substantial variance between replicates and significant differences between treatments (Figure 6). Therefore, the results are more likely due to *B*. *subtilis* colonization than nutrient acquisition via *B*. *subtilis* digestion. This variance is also consistent with previous work examining bacterial growth in *C. elegans*, where stochasticity has a significant effect on bacterial abundance (Vega & Gore, 2017). Finally, a recent study has shown that *B*. *subtilis* extends *C. elegans *lifespan postheat shock through the production of biofilm (Donato et al., 2017), demonstrating that live cells are present in and actively colonizing the host gut. Taken together, these results suggest that the fitness benefits conferred by *B*. *subtilis* postheat shock is likely largely due to host–microbe interactions within the host.

Our study demonstrates that interacting with the appropriate microbe under stressful conditions can benefit hosts in terms of reproduction, which could have significant implications for host population growth and evolution in the long term. This could select for association with the microbe in future generations, leading to the potential for coevolution of the partners within the framework of a mutualistic symbiosis. Importantly, the mechanistic nature of the beneficial interaction between *C. elegans* and *B*. *subtilis* may not dictate the system's capacity for mutualistic coevolution. Studies have shown that certain bacteria may serve roles in *C. elegans *distinguishable from diet (Berg et al., 2016; Cabreiro & Gems, 2013; Dirksen et al., 2016; Gerbaba, Green‐Harrison, & Buret, 2017; Zhang et al., 2017). Because *B*. *subtilis* can survive within the host, the host and microbe have the potential for mutualistic coevolution when their fitness aligns. However, even if the fitness differences we observed are due to nutrients obtained via *B*. *subtilis* digestion and not due to the impact of having maintained association with live bacteria, then mutualistic coevolution is still possible. For example, leaf‐cutter ants and the fungi they cultivate have been coevolving with each other for millions of years, even though the fungi serve primarily as the ant's food source (Schultz & Brady, 2008; Weber, 1966). Furthermore, as evident in extant models of symbiosis (Davitt, Chen, & Rudgers, 2011; Heath & Tiffin, 2007; McMullen, Peterson, Forst, Blair, & Stock, 2017; Vorburger, Ganesanandamoorthy, & Kwiatkowski, 2013; Weldon et al., 2013), the *B. subtilis–C. elegans *interaction is likely context‐dependent: fitness benefits are obtained optimally only under certain environments (e.g., heat stress), at a certain stage in the host's life cycle, and with the right microbe. While more work is necessary to determine the mechanism by which *B*. *subtilis* increases *C. elegans *fecundity after heat stress, our work provides further evidence for the critical role that bacteria can play in the evolution and ecology of their hosts.

## CONFLICT OF INTEREST

The authors declare no competing interests.

## AUTHOR CONTRIBUTIONS

KLH, NMG, and LTM designed the study. KLH performed the experiments. KLH and LTM analyzed the data. KLH wrote the manuscript with input from NMG and LTM.

## Data Availability

All relevant data are available at the Dryad Digital Repository (https://doi.org/10.5061/dryad.7kc589v).
